# Three Cases of Rare Dislocation

**Published:** 1873-01-01

**Authors:** N. Senn

**Affiliations:** Ashford, Wis.


### Original Communications.

### THREE CASES OF RARE DISLOCATION.

BY N. SENN, M. D., ASHFORD, WIS.

*Read before the Rock River Medical Society.*

The majority of malpractice suits arise on account of viciously united fractures, or unreduced dislocations.

Not only law renders these injuries of great interest and anxiety to the surgeon, but it is likewise an everlasting torture to his professional pride to witness his former patients walk lame, with deformed limbs, traveling advertisements of misfortune, neglect, or ignorance.

Bad results, in such cases, may generally be traced to one of three causes, viz.: 1st, The very nature of the injury itself; 2d, Non-co-operation on the part of the patient during treatment; 3d, Want of proper skill or care on the part of the medical attendant himself. For the first two we are not responsible; and, as to the last, I trust that none of us feel ourselves guilty.

Cases do occasionally occur which baffle our skill; where, on our part, most that can be done is simply to be an idle spectator, while nature completes the hideous work as well as she may. Such cases are of great importance, and deserve to be made public among the members of our profession. If they do not add to individual reputation, they serve well for the mutual benefit of us all.

Three cases of dislocations have lately occurred in my practice, which well deserve to be called rare.

The two first I report on account of their incurability, from the character of the injuries; the last on account of its extreme unfrequency, and the special interest attached to it, as it occurred to a professional brother.

Case I.—*Anterior Dislocation of Sternal end of Left Clavicle:* J. H., æt. 10, fell about ten feet from a barn floor, falling on the shoulder of the affected side. He was unable to use the arm after the injury. Twenty-four hours after the accident I examined him, and found the arm in the same position as in fracture of the clavicle: the sternal end of the clavicle formed a conspicuous protuberance in front of the sternum; its articular surface could be distinctly defined through the skin covering it. The pain was not severe, and limited to the seat of the dislocation.

By seating the patient on a chair and placing my knee against the spine, drawing back the shoulder—having at the same time direct pressure made—I was able to effect complete reduction. As soon as the traction on the shoulder was removed, the bone escaped from the articular surface to its abnormal position. Of the different dressings applied, none of them was sufficient to retain it in its place. I therefore became disgusted with them all, and abandoned everything else except two strips of adhesive plaster, applied in the same manner as for fracture of the clavicle. At the same time, I informed the parents that the deformity would remain, but that in all probability the utility of the shoulder would not become impaired.

In two weeks I removed all dressings and placed the arm in a sling. At present, about two months after the accident, the deformity remains nearly as conspicuous as when he first applied for advice, but the function of the arm and shoulder is perfect.

Hamilton has seen seven cases of this dislocation; in one, after repeated trials, he failed to reduce the bone; and he freely confesses that in none of them was the retention completely effected. I believe that in most of these cases the anterior edge of the articular surface of the sternum is chipped away by the dislocating force acting through the medium of the clavicle, rendering retention extremely difficult, if not impossible.

Case II.—*Dislocation of Femur, with Fracture of Rim of Acetabulum:* J. I., æt. 22, a strong, muscular man, was injured May 21st, 1872, while he was pushing a hand-car before him. Another one coming from behind with greater speed struck him just above the great trochanter, on the side of the pelvis, while the limb was in nearly a straight position. The force was applied from above downward. The patient was unable to walk or move his limb without creating great pain. A physician was called, soon after, who diagnosed a simple dorsal dislocation of the femur, which, according to his statements, he reduced very easily by slight manipulation. He saw the patient several times subsequently, and pronounced everything as "all right." For a number of days the patient suffered great pain in the region of the joint.

In a few weeks he attempted to walk, but found it impossible to rest the weight of the body on the affected limb.

Two months after the accident he sought my advice. I found the limb two inches shorter than the opposite one; the head of the femur could be distinctly felt on the dorsum ilii; the foot and knee inverted. All movements were limited. The patient being very anxious that at least an attempt should be made at reduction, I operated, with the assistance of Drs. Loehr and Lueck. We first tried manipulation, according to the directions given by Dr. Reid, and Profs. Bigelow and Gunn, but none yielded the desired result. We next made use of extension, by a number of assistants, while the patient was profoundly under the influence of ether, with the result of changing somewhat the location of the head of the bone. After a number of attempts, we desisted. The patient was put in bed, and extension applied by means of weight and pulley. No unfavorable symptoms followed.

A week after, we put the patient under the influence of ether, and again resorted to manipulation, and again failed. We now applied the pulley, the thigh being bent across the opposite one, and rotated inwards. In this direction extension was applied. After using a great deal of force the head of the femur was found to move, and, with an audible noise, advance nearly two inches. No justifiable force could bring it any farther forward.

On close measurement the limb was found of normal length, and as long as held in this position, rotation, adduction and abduction were easily made; but as soon as extension was entirely removed, and especially if the leg was moved at the same time, the bone slipped back to its old location, accompanied by the same rough noise. This was repeated several times, with the same results. I now became suspicious that there must be something wrong about the acetabulum, and informed my colleagues accordingly. I was now able, with my own force, to bring the limb where it had previously required the pulley, when I brought it down to its normal length. I could feel, by rotating it, the smooth surface of the acetabulum. About a quarter of an inch, posteriorly, it seemed to pass over a rough edge, and then, without any further obstacle, receded to its old place. Manipulation would now bring it in the same position without any better result following.

We were now all satisfied that there existed, with the dislocation, a fracture of the posterior and upper portion of the rim of the acetabulum.

We brought the limb into its natural position and re-applied extension by the weight and pulley; a pelvic band finished the dressing.

The limb remained in this position for over a week, when, unfortunately, the extension apparatus got out of order. The patient failed to notify the attending physician immediately, and two days after he found an inch shortening. Extension was again applied. About three weeks after the operation I saw him, and found over an inch shortening, with preternatural prominence of the great trochanter.

The treatment will be continued for some time longer, but I am inclined to think that eventually the bone will return to the place it occupied before the operation.

This injury we would naturally expect to be a very infrequent one. Some of the most extensive surgical text-books do not even refer to it. Chelius mentions it, but gives a very indefinite description. Richter, in his special work on "Fractures and Dislocations," published in 1833, page 126, says: "In fractures of the cotyloid cavity it is said that the head of the femur is drawn upward, and the great trochanter forward, producing shortening of the limb and inversion of the foot and knee, symptoms which easily might be mistaken for a dislocation." To effect reposition, he recommends extension and counter-extension. To insure retention, he directs to keep both limbs in apposition, upon a double inclined plane.

Erichson (page 270) believes that fractures of the acetabulum can only occur from great violence, directly applied to the hip. He regards the result always as unfavorable, if dislocation has taken place.

Ashhurst, in his late work on surgery (page 242), believes, with Dr. Bigelow, that fracture of the acetabulum, complicating dislocation, is less frequent than is generally supposed, and should never be regarded as such, unless crepitus is felt. Another diagnostic symptom he mentions, viz.: Easy reduction and difficult retention.

Bardeleben (vol. II., page 407) says: "In fractures of the acetabulum there almost always co-exists a dislocation of the femur, which is easily reduced, accompanied by crepitus, but is retained with great difficulty." For treatment, he considers permanent extension in a semi-flexed and abducted position essential to prevent re-dislocation.

Hamilton, in his work on "Fractures and Dislocations," gives an excellent description of several cases, but in all, re-dislocation eventually took place. For treatment he recommends permanent extension and a pelvic band.

The diagnosis in this case could not be made before the operation, on account of the time which had elapsed since the injury had been received. The physician first in attendance undoubtedly reduced the dislocation, but overlooked the complication.

In recent cases it may be distinguished from a simple dislocation: 1st, from the ease by which the reduction is made and the difficulty of retention; 2d, the presence of crepitus; 3d, the manner in which the dislocating force was applied, and the position of the limb at the time it was received. A dislocation occurs only, according to Prof. Gunn, when the limb is not in an axis with the body. The contrary is generally true of fractures of the acetabulum.

Although treatment appears to be of doubtful benefit, an attempt should be made to keep the parts in apposition until the acetabulum has gained sufficient strength to support the weight of the body. Bony union between the fractured ends, if not impossible, is at least improbable, from the anatomical relation of the parts.

CASE III.—*Dislocation of Tibia and Fibula forward:* Dr. L., æt. 36, a tall, muscular man, on August 22d, 1872, while turning a corner upset his buggy, and was thrown out. Just how he fell, or the exact manner in which the injury was received, he was unable to state. As there were marks of violence in the popliteal space and in front of the ankle, I am inclined to think that his leg was caught between the spokes of the wheel. He was unable to stand on his right leg, or move it without intense pain. He also noticed the great deformity which too plainly demonstrated to him that something serious had taken place. Being accidentally in the neighborhood, the messenger met me on the road, and I saw him about an hour after the accident. He suffered considerably from the shock; the features were pale, and expressive of a peculiar indifference of what had happened; surface covered with a cold, clammy perspiration; extremities cold; pulse feeble and frequent. The limb presented a characteristic appearance; it was flexed, and the foot slightly inverted; shortening, amounting to three inches; the antero-posterior diameter of the knee was greatly increased, the head of the tibia and patella forming a sharp protuberance in front, while the condyles of the femur formed a roundish prominence in the popliteal space. The articular surface of the tibia could be readily distinguished through the skin. The pulsations of the posterior tibial artery were hardly perceptible, and the whole leg felt cold. Reduction was accomplished by making counter-extension and extension by a number of assistants; the former just above the knee, and the latter at the ankle, while the limb was flexed at about an angle of forty-five degrees. I made direct pressure upon the bones. Considerable force was required to dislodge the bone, when it suddenly—with a loud snap— was restored to its normal position. The limb was placed upon a posterior splint, and bladders, filled with pounded ice, applied to the joint. Considerable swelling took place during the first night and remained for three days, when it gradually subsided. At no time was the pain severe. The ice was continued for the first three days, and afterward, followed by tinc. opii and aq. plumbi. He had often queer sensations along the course of the peroneal nerve; and several points on the foot, supplied by it, were without sensation. In six weeks he was able to walk around with one crutch, but the anterior and peroneal muscles were found almost completely paralyzed, causing a position of the foot analogous to equino varus. Undoubtedly, at the time the injury was received, the peroneal nerve was injured, giving rise to this unpleasant result. Electricity and shampooing were perseveringly used with the hope of finally restoring contractility to the affected muscles. At present the knee is perfectly well, and the patient is able to walk well with the aid of a cane, the paralysis having considerably improved.

Velpeau gathered thirteen cases of anterior dislocation of the tibia and fibula. Hamilton gives the history of five other cases, none of which he seems to have seen himself. From these facts we may presume that it must be an accident of very unfrequent occurrence.

Almost perfect recovery followed in most of the cases, in from six weeks to three months.

